# Reconstruction after hemisacrectomy with a novel 3D-printed modular hemisacrum implant in sacral giant cell tumor of the bone

**DOI:** 10.3389/fbioe.2023.1155470

**Published:** 2023-05-02

**Authors:** Zhaorui Lv, Jianmin Li, Zhiping Yang, Xin Li, Qiang Yang, Zhenfeng Li

**Affiliations:** ^1^ Department of Orthopedics, Qilu Hospital of Shandong University, Jinan, Shandong, China; ^2^ Cheeloo College of Medicine, Shandong University, Jinan, Shandong, China

**Keywords:** 3D-printed, implant, hemisacrectomy, reconstruction, giant cell tumor of the bone

## Abstract

**Background:** There are a limited but increasing number of case reports and series describing the use of 3D-printed prostheses in bone tumor surgery.

**Methods:** We describe a new approach to performing nerve-preserving hemisacrectomy in patients with sacral giant cell tumors with reconstruction using a novel 3D-printed patient-specific modular prosthesis. The series included four female and two male patients with a mean age of 34 years (range, 28–42 years). Surgical data, imaging assessments, tumor and functional status, implant status, and complications were retrospectively analyzed in six consecutive patients.

**Results:** In all cases, the tumor was removed by sagittal hemisacrectomy, and the prosthesis was successfully implanted. The mean follow-up time was 25 months (range, 15–32 months). All patients in this report achieved successful surgical outcomes and symptomatic relief without significant complications. Clinical and radiological follow-up showed good results in all cases. The mean MSTS score was 27.2 (range, 26–28). The average VAS was 1 (range, 0–2). No structural failures or deep infections were detected in this study at the time of follow-up. All patients had good neurological function. Two cases had superficial wound complications. Bone fusion was good with a mean fusion time of 3.5 months (range, 3–5 months).

**Conclusion:** These cases describe the successful use of custom 3D-printed prostheses for reconstruction after sagittal nerve-sparing hemisacrectomy with excellent clinical outcomes, osseointegration, and durability.

## Introduction

Giant cell tumor (GCT) is a primary bone tumor with local aggressiveness, accounting for 5% of all primary bone tumors and occurring in young adults (Mendenhall et al., 2006). The treatment of sacral giant cell tumors is challenging because of their complex anatomy, overload, extensive defects, sacral nerve root involvement, and a higher rate of local recurrence than any other skeletal site (Shen et al., 2012). Vertical resection of the tumor in half of the sacrum disrupts the spinal–pelvic continuity and requires reconstruction to restore bony connections and mechanical transmission (Clarke et al., 2014; Reynolds et al., 2016). Tumors involving half of the sacrum are rare, and standard protocols for resection and reconstruction are lacking (Palejwala et al., 2017). Spine–pelvis reconstruction usually relies on various forms of internal fixation, such as nail rods, titanium mesh, and long bone segment bone graft; however, due to the small contact area and limited three-dimensional configuration, it is difficult to meet the requirements (Bederman et al., 2014).

The ideal reconstruction method should include three aspects of spondylopelvic fixation, posterior pelvic ring fixation, and anterior spinal support, which are still difficult to achieve satisfactorily with conventional techniques considering the anatomical and weight-bearing characteristics of the lumbopelvic junction (Bederman et al., 2014). The incidence of failure of internal fixation devices, such as non-healing bone grafts, internal fixation displacement, fracture, and loosening, is high (Tang et al., 2018). The conventional sacral reconstruction method remains suboptimal, and prolonged bed rest is usually required.

With the development of digital orthopedic technology, more and more patients are using personalized prostheses for complex orthopedic reconstructions. The problem can be solved using 3D-printed prostheses, with the advantages of conformal matching and osseointegration techniques (Tong et al., 2020). Dong Ah Shin reported a 3D-printed one-piece hemisacral prosthesis for the reconstruction of bone defects after hemisacral resection for sacral osteosarcoma (Kim et al., 2017). If the sacral nerve is preserved, the one-piece prosthesis is difficult to be placed by nerve obstruction. Based on 3D printing technology, our center has applied 3D-printed modular prostheses that can coexist with the sacral nerve for sacral reconstruction since 2017 with satisfactory results (Lv et al., 2020).

The use of 3D-printed prostheses for hemisacral reconstruction of sacral GCT has not been previously reported. To overcome the limitations of traditional reconstruction, we innovatively used 3D printing technology for the preoperative design and successfully reconstructed the bone defect after hemisacral resection for sacral GCT with a 3D-printed hemisacral prosthesis. The purpose of the study was to investigate the tumor resection techniques, reconstruction strategy, and postoperative function outcomes of 3D-printed prostheses for hemisacral stability reconstruction.

## Materials and methods

### Patients

The study was approved by the Ethics Committee of Qilu Hospital, Shandong University, and all patients signed an informed consent document. Six patients, two male and four female, with sacral GCT involving half of the sacrum admitted from 2018 to 2021 with a mean age of 34 years (range, 28–42 years), were included. All patients complained of persistent occult pain in the sacrococcygeal region, and two cases had progressive sacral nerve compression symptoms. All patients underwent a preoperative X-ray, CT, and MRI of the pelvis. Four cases involved S1–S3, and two cases involved S1–S2. All patients underwent preoperative puncture biopsy to clarify the pathological diagnosis.

### The 3D implant

**FIGURE 1:**
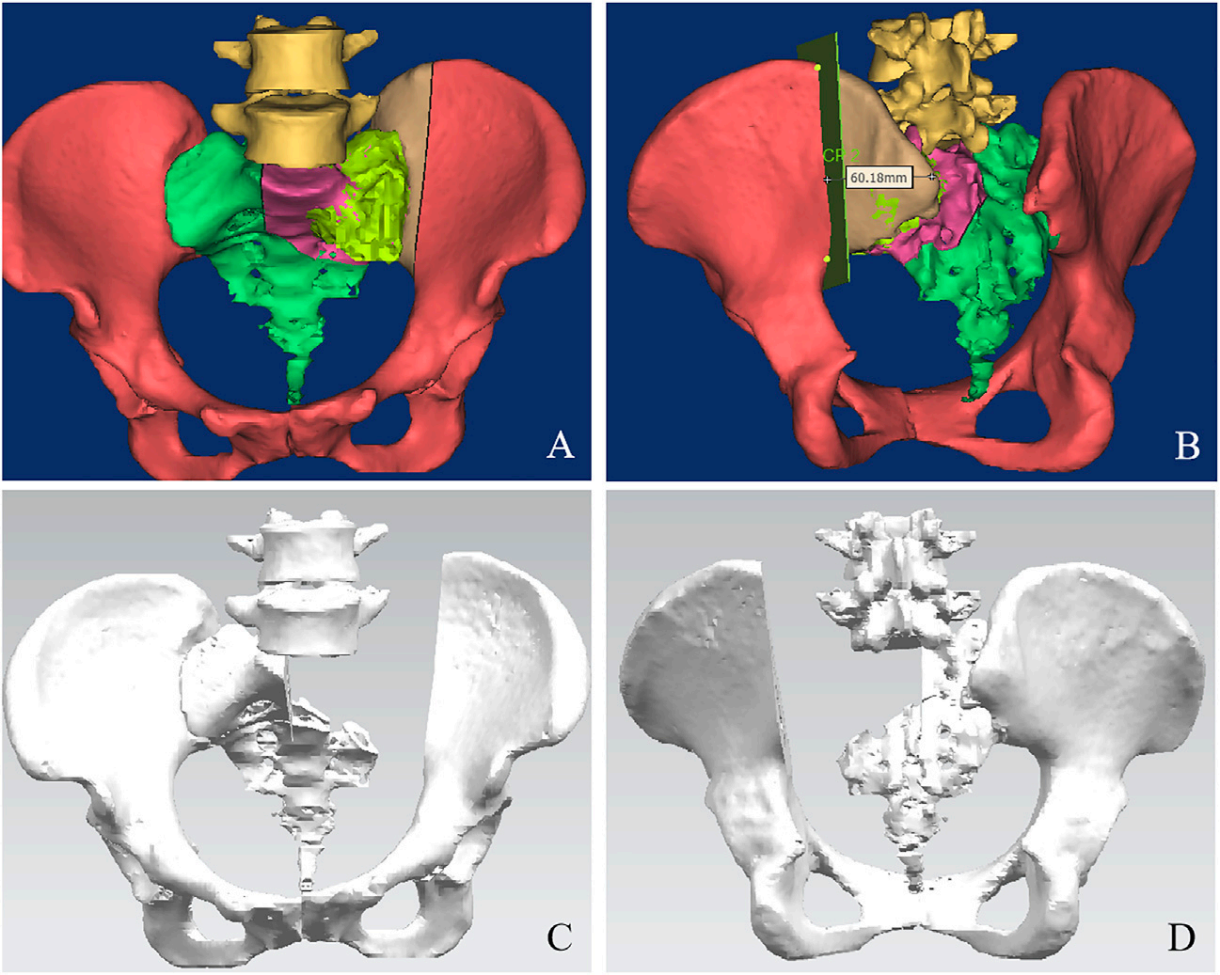
A 3D bone tumor model from CT data was created for surgical planning **(A, B)**. Bone defect model after tumor resection **(C, D)**.

**FIGURE 2:**
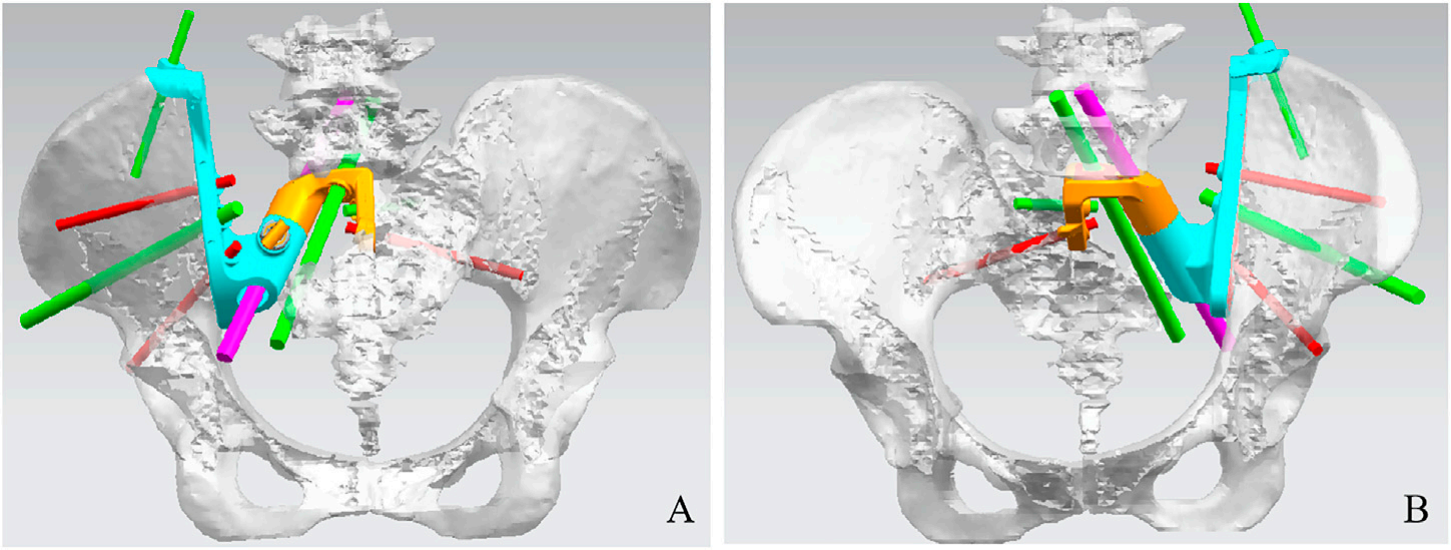
Design of the sacral implant. Dorsal view **(A)** and front view **(B)** of the sacral implant 3D model.

**FIGURE 3:**
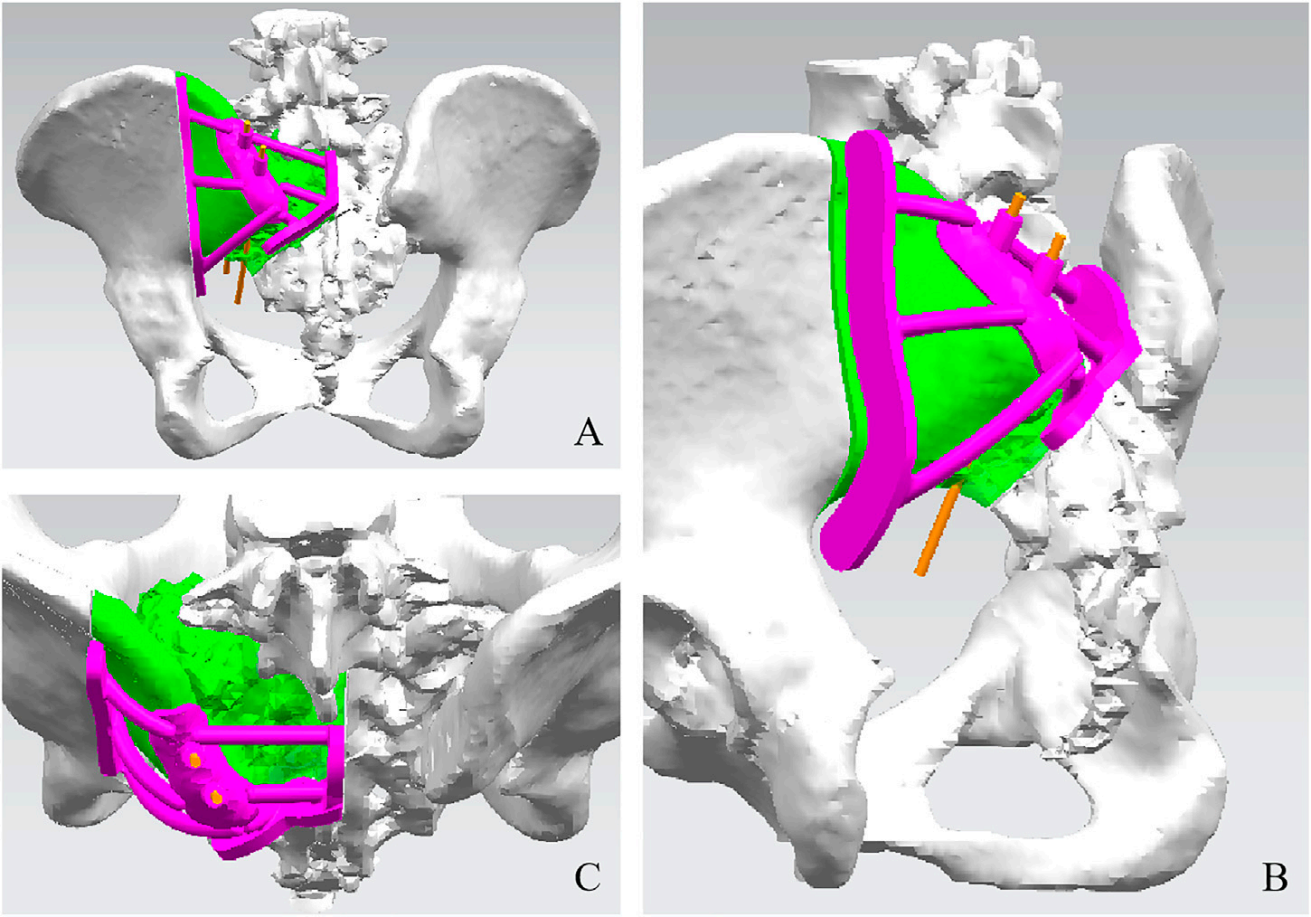
Design of the cutting guide. Dorsal view **(A)**, side view **(B)**, and front view **(C)** of the sacral implant 3D model.

The pelvis CT images were stored in the DICOM format. Medical image processing software, Mimics (Materialise, Belgium), was used to reconstruct the CT images of the patient’s pelvis and obtain a model of the bone defect after simulated osteotomy (Figure 1). The prosthesis was designed according to the shape of the patient’s bone defect and stress transmission (Figure 2). The connection between the iliac wing fixation and the main body is a rotatable sleeve, which is locked with screws. The prosthesis is screwed to the fifth lumbar vertebra, the residual sacrum, and the ilium. A universal screw head is designed on both sides of the dorsal surface, which can be connected to a titanium rod for further fixation with the lumbar pedicle screw. The prosthesis–bone interface is a loose and porous trabecular structure, which is conducive to osseointegration. The surface of the prosthesis adjacent to the sacral nerve is smooth to reduce irritation of the nerve. The rough sprayed surface and small holes around the perimeter of the prosthesis facilitate the wrapping and fixation of the surrounding soft tissues. The porous metal trabecular structure with a porosity of 70%–80% and a size of 600–700 μm is produced by 3D printing technology, and the osteotomy guide is designed and produced according to the osteotomy direction, osteotomy angle, bone surface morphology at the osteotomy location, and the position and angle of the kerfing needle (Figure 3).

### Surgical procedure

To reduce intraoperative hemorrhage, all patients underwent preoperative denosumab therapy (weekly for the first month). During surgery, general anesthesia was administered in the prone position. The surgical incision was a posterior median curvilinear incision. A temporary blocking of the abdominal aortic balloon was performed to reduce bleeding. The left dorsal surface of the sacrum, the affected sacroiliac joint, and the posterior structures of the L4–L5 lumbar vertebrae were revealed by successive incisions. The dural sac and sacral nerve were revealed using an ultrasonic bone knife, and the L5 nerve root was protected. The tumor was resected in pieces, preserving the sacral nerve roots as much as possible. The osteotomy guide was placed in the appropriate area, and the ultrasonic osteotome was used under the direction of the osteotomy guide to resect half of the L5/S1 intervertebral disc using iliac osteotomy and sacral sagittal osteotomy, ensuring that no tumor remains and that adequate hemostasis is achieved. The prosthesis was placed in the bone defect area and attached to the iliac osteotomy, the lower L5 endplate, and the residual sacrum. The two parts of the prosthesis were connected by a sleeve, which was precisely matched and fixed. It was fixed to the L5 vertebral body and the iliac bone by means of screws. A metal rod was used to connect the lumbar spine posteriorly to the prosthesis. Soft tissue sutures were fixed to the holes on the surface of the prosthesis. Intraoperative fluoroscopy was performed to see that the prosthesis was installed in accordance with preoperative planning. The incision was closed layer by layer after thorough hemostasis and irrigation with the placement of drainage tubes.

### Postoperative management and follow-up

Postoperative antibiotics were used to prevent infection. Measures were taken to prevent deep vein thrombosis. Blood transfusion was given if necessary. Symptomatic treatment such as nerve nutrition was given. Functional bladder exercises were performed from 3 days postoperatively, and removal of the catheter was attempted 2 weeks postoperatively when it was changed. The drainage tube was usually kept until the drainage flow was less than 50 mL/24 h. Progressive functional exercise of the lower extremities was usually performed in bed for at least 4 weeks with brace-assisted support. After discharge, the patient was followed up regularly through outpatient clinics, with physical and imaging examinations every 3 months for the first 2 years after surgery and every 6 months thereafter. Efficacy evaluation indicators include oncological prognosis, sacral nerve and limb function, and prosthetic osseointegration and complications. The results of the limb function were evaluated using the MSTS score at the final follow-up.

## Results

**FIGURE 4:**
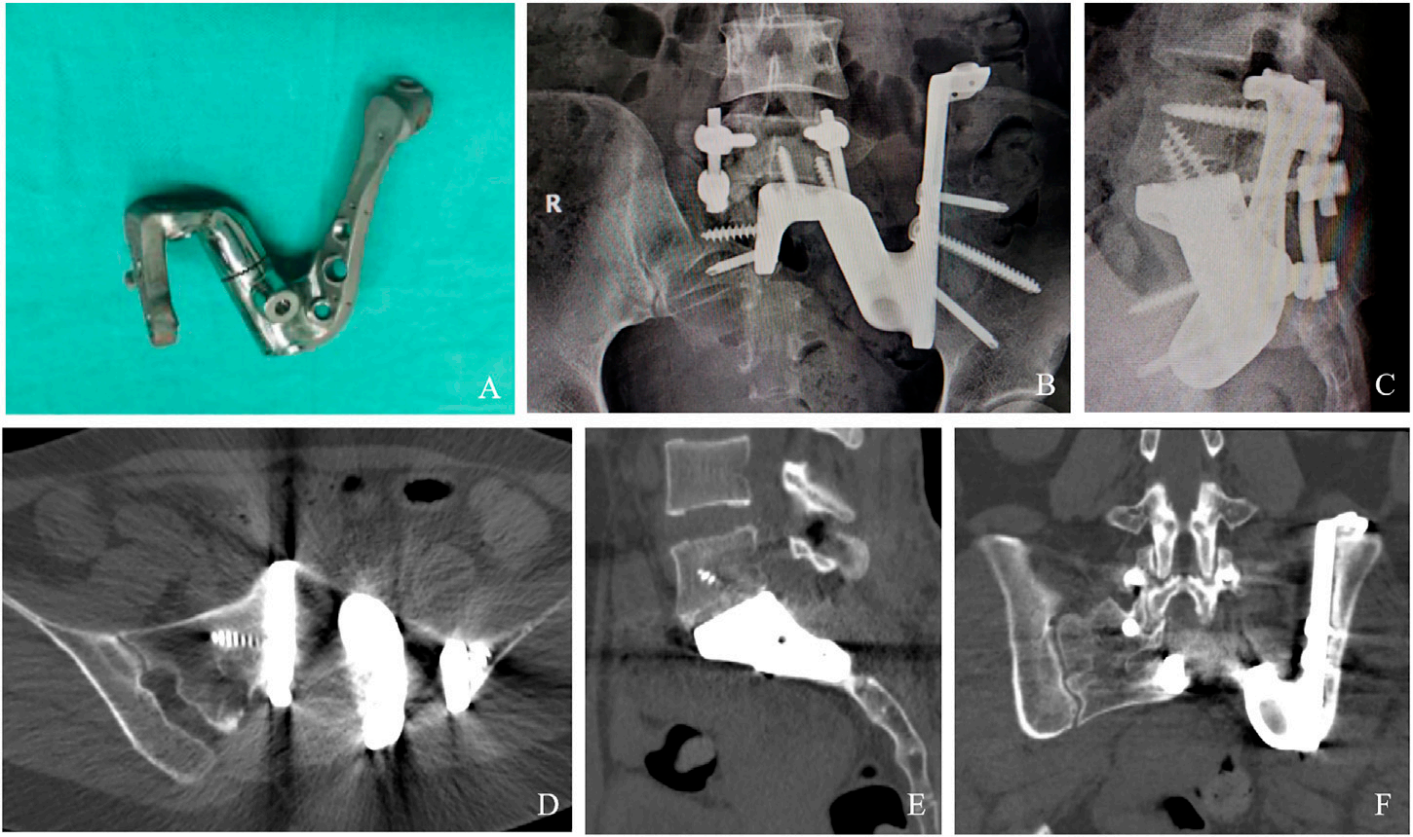
Results of prosthesis implantation. Physical object of the implant **(A)**. X-ray examination 3 years after operation **(B, C)**. CT showing excellent osseointegration at the bone–implant junctions in axial **(D)**, sagittal **(E)**, and coronal **(F)** views.

A relatively satisfactory surgical border was obtained by completing tumor resection in blocks and custom 3D-printed sacral prosthesis reconstruction according to the preoperative design, with maximum preservation of sacral nerve function (Table 1). The average operative time and intraoperative blood loss were 460 min (range, 360–580 min) and 3,450 mL (range, 3,000–5,000 mL), respectively. No serious intraoperative complications or deaths occurred. Two patients had wound problems. These wound problems were successfully resolved by debridement, drainage, and antibiotics. No deep infections occurred. The mean follow-up was 25 months (range, 15–35 months). At the last follow-up, all patients survived disease-free. No patient had local recurrence or metastasis. No or only slight loss of the sphincter function was noted in all patients. All patients had no fecal incontinence, and the catheter was successfully removed within 3 weeks after surgery. All patients were able to walk postoperatively. At the 3-month postoperative follow-up, all patients were able to walk without assistance and had no gait disturbances (Supplementary Material). The average VAS was 1 (range, 0–2). The mean MSTS score was 27.2 (range, 26–28). Radiographs of the patients showed that the 3D-printed prosthesis was well positioned without loosening, sinking, or displacement, and there were no internal fixation failures, such as broken nails or rods (Figure 4). CT showed complete fusion of the prosthesis with the ilium and lumbar spine (Figure 4), with a mean fusion time of 3.5 months (range, 3–5 months).

**TABLE 1:** Surgical characteristics and outcomes.

Case	Age	Sex	Tumor level	Operative time (min)	Blood loss (mL)	Follow-up (month)	Neural status	Time to union (month)	VAS score	MSTS	Complications
1	28	F	S1–3	420	3,000	30	Intact	4	1	28	None
2	34	F	S1–3	580	5,000	32	Intact	5	2	26	Wound dehiscence
3	36	F	S1–3	360	3,500	28	Intact	3	1	27	None
4	42	M	S1–2	500	3,000	25	Intact	3	0	28	None
5	33	F	S1–3	400	3,200	20	Intact	3	1	27	Wound dehiscence
6	31	M	S1–2	500	3,000	15	Intact	3	1	27	None

## Discussion

To the best of our knowledge, the use of a custom 3D-printed sacral prosthesis for lumbosacral reconstruction is extremely rare (Houdek et al., 2020). Spinal–pelvic reconstruction after hemisacrectomy is a challenging and high-risk task (Palejwala et al., 2017). In recent years, 3D printing technology has been successfully applied to preoperative planning, resection, and reconstruction of bone tumor surgery, opening up new routes for reconstruction after the resection of bone tumors in complex sites (Habib et al., 2022). In this group of cases, we used a new 3D-printed prosthesis that precisely matches the bone defect at the complex site and uses a winged structure to maximize bone contact and reduce vertical stresses, thereby creating sufficient immediate biomechanical stability in the area. The prosthesis is easy to install intraoperatively, eliminating the need to trim and install staples, titanium mesh, and allograft bone, simplifying the sacral reconstruction steps, and reducing operative time, bleeding, and intraoperative fluoroscopy. The porous structure of the prosthesis facilitates osseointegration and provides the unique, long-term stability advantages of promoting adjacent bone fusion and reducing the rate of internal fixation failure (Mayfield et al., 2022). The design of the small holes around the implant facilitates soft tissue reconstruction. These features may have contributed to the successful healing of this area in the patients.

Osseointegration is essential for the long-term stability of the prosthesis (Yuan et al., 2022); otherwise, aseptic loosening is inevitable. Follow-up results showed that good osseointegration could be achieved at the bone–implant interface, indicating that this 3D-printed porous implant is highly histocompatible and fully meets the complex mechanical environment of the lumbosacral region. The prosthesis offers unique long-term stability advantages and low internal fixation failure rates, with no patients experiencing prosthetic loosening and broken nails. 3D-printed prostheses must be placed precisely as planned. The prosthesis is used in conjunction with patient-specific osteotomy guides to ensure that the bone defect is matched to the prosthesis and that unnecessary soft tissue exposure is minimized. To promote osseointegration, autogenous bone grafts are inserted intraoperatively into the suture at the bone–implant interface (Wang et al., 2020). Timely weight-bearing can promote osteogenesis, but excessive early weight-bearing should be avoided. It is most important to achieve adequate initial stability using an anatomically correct prosthesis and well-positioned screws.

Sacral nerve-preserving fractional resection of sacral osteoblastic giant cell tumors has achieved satisfactory local control and is now widely accepted (Wang et al., 2020). We have preserved the sacral nerve as much as possible in patients with sacral osteoblastic giant cell tumors, which is critical for maintaining good physical and mental health, bowel function, and sexual function. Most current sacral prostheses are one-piece designs, and if the sacral nerve is preserved, the prosthesis is difficult to place due to nerve obstruction. A custom 3D-printed split full sacral prosthesis is a prosthesis that can coexist with the sacral nerve. The split design of the prosthesis allows the prosthesis to safely pass through the sacral nerve, avoiding excessive strain on the nerve.

Control of intraoperative bleeding is an important safeguard for completing surgery for sacral giant cell tumors of the bone (GCTB). With the use of bleeding control drugs, preoperative selective arterial embolization, and intraoperative aortic balloon, nerve-preserving surgery for sacral GCTB has become feasible and the risk of recurrence is acceptable. In this study, neoadjuvant treatment with a short course of preoperative denosumab reduced tumor blood supply without increasing tumor–nerve adhesions (Liang et al., 2022). Abdominal aortic balloon placement on the day of surgery can provide satisfactory bleeding control. A clear surgical field helps separate and protect the sacral nerve, which improves the safety and cure rate of surgery.

Surgery for sacral tumors carries a high risk of complications. Incisional complications (infection or dehiscence) have been reported in 29.2% of sacral tumor patients (Li et al., 2013). For this, our experience is that deep fascial layers are tightly closed to reduce dead space, adequate drainage is placed, and systemic antibiotics are used to prevent local infection. The incision is changed promptly to avoid contamination by feces. The characteristics of trabecular drainage fluid are recorded, and the microbiological culture is promptly performed if the trabecular drainage fluid is cloudy or if the patient has a fever of >38.5°C.

The 3D hemisacral prosthesis achieved more satisfactory clinical results in the reconstruction of stability after the resection of sacral GCT. This study also has limitations: as a retrospective study, selection bias is inevitable, and the follow-up period is relatively short. Due to the low incidence of the disease and the relatively small number of cases in this group, the incidence of complications may be underestimated. Future studies should compare the effects of different reconstruction modalities on the reconstruction outcome, which needs to be evaluated using clinical studies with a higher level of evidence.

## Conclusion

The prosthesis has achieved more satisfactory clinical results in hemisacral osteotomy stability reconstruction, allowing patients to move early, reducing postoperative complications, and improving quality of life, which is worthy of further promotion in clinical practice .

## Data Availability

The original contributions presented in the study are included in the article/Supplementary Material; further inquiries can be directed to the corresponding author.
